# Torsional and burst mode phacoemulsification for patients with hard nuclear cataract: A randomized control study

**DOI:** 10.1097/MD.0000000000015870

**Published:** 2019-05-31

**Authors:** Wan-Ju Yang, Xing-Hua Wang, Fang Zhao, Zhong-Ming Mei, Shuang Li, Yi Xiang

**Affiliations:** a Department of Ophthalmology, Central Hospital of Wuhan, Tongji Medical College of Huazhong University of Science and Technology; b Department of Ophthalmology, Union Hospital, Tongji Medical College, Huazhong University of Science and Technology, Wuhan, Hubei Province, China.

**Keywords:** burst mode, cataract, phacoemulsification, torsional mode, trypan blue

## Abstract

This article aims to evaluate the outcomes of torsional and burst mode phacoemulsification in hard nuclear cataracts.

Eighty eyes with grade IV or V nuclear opalescence were treated with phacoemulsification and intraocular lens implantation using conventional mode (Group A, n = 40) or torsional and burst mode phacoemulsification (Group B, n = 40). For good visualization of anterior capsule, trypan blue was injected to the anterior chamber before continuous circular capsulorhexis. The mean cumulative dissipated energy and ultrasound time were recorded. The best-corrected visual acuity, endothelial cell density, and central corneal thickness were measured before and at 1 month after surgery.

The cumulative dissipated energy and ultrasound time of Group B were significantly less than that of Group A. The postoperative best-corrected visual acuities of the 2 groups were comparable. At 1 month after surgery, the changes in the endothelial cell density were significantly greater in Group A than in Group B, and the changes in the central corneal thickness were not significantly different between the 2 groups.

Torsional and burst mode is a safe and effective surgical method for treating hard cataracts.

## 1 Introduction

With improvements in the techniques of cataract surgery, phacoemulsification has recently been widely used by ophthalmologists for cataract surgery with increased safety and efficacy.^[1–5]^ However, mature or hypermature cataract is common in China, due to restricted medical or poor economic conditions. The application of high-intensity ultrasound energy during phacoemulsification can easily lead to tissue damage and increases the surgical risk of treating mature or hypermature cataract. Postoperative complications such as corneal edema, anterior chamber fibrin exudation, and long recovery time occur more frequently.

Torsional ultrasound devices have been reported to reduce operative complications and improve visual outcomes in patients undergoing cataract surgery.^[6]^ In the conventional ultrasound mode, the phacoemulsification tip moves forward and back at a high frequency, producing a repulsion effect. Ozil torsional mode, a new ultrasound mode introduced by the Infiniti Vision System phacoemulsification instrument (Alcon Laboratories, Fort Worth, Texas), is more efficient than the conventional mode for phacoemulsification surgery treating hard nuclear cataract.^[1,2]^ In addition, burst mode, an ultrasonic power mode introduced in recent years, is used to reduce the mean ultrasound time (UST) and cumulative dissipated energy (CDE), and is more suitable for hard nuclear cataract compared with continuous mode.^[3–5]^

Continuous circular capsulorhexis (CCC) is a procedure that is widely used during cataract surgery. However, it is difficult to use CCC in the treatment of hard or mature cataract, due to the poor visibility of the anterior capsule. Fortunately, the visibility of the anterior capsule can be improved when performing capsulorhexis via injection of trypan blue into the anterior chamber with a small amount of viscoelastic agent, thus making CCC easier for the treatment of hard or mature cataract.^[7]^

The present study compared the torsional and burst mode combined with trypan blue staining, to that of the conventional mode, for phacoemulsification treating 80 patients (80 eyes) with hard cataracts, specifically in terms of clinical efficacy, safety, and postoperative clinical outcomes.

## 2 Materials and methods

### 2.1 Patients

This study was performed at the Department of Ophthalmology, Wuhan Central Hospital, Tongji Medical College of Huazhong University of Science and Technology. The Ethics Committee of Wuhan Central Hospital, Tongji Medical College of Huazhong University of Science and Technology, approved this study, and informed consent was obtained from each patient. The study was conducted in accordance with the tenets of the Declaration of Helsinki.

This clinical study enrolled 80 patients (80 eyes) who had received diagnoses of cataract at the Department of Ophthalmology, Wuhan Central Hospital, Tongji Medical College of Huazhong University of Science and Technology, from January 2015 to December 2016. All patients who had nuclear opalescence of Grade IV or V and a history of progressive decrease in visual acuity were recruited. Cataract was graded according to the Lens Opacities Classification System III.^[8]^ Patients were excluded if they had white cataracts, a history of ocular surgery, or eye diseases such as corneal pathology, Fuchs’ dystrophy, uveitis, and glaucoma or systemic diseases with eye involvement. Patients with any of the following eye conditions were excluded from this study: intraocular pressure (IOP) > 21 mm Hg; uveitis with active inflammation; a retinal detachment or suspected retinal detachment on ocular ultrasonography; corneal disease; traumatic cataract; or ocular or systemic diseases that could influence corneal endothelial cell function.

The patients were randomly assigned to undergo phacoemulsification and intraocular lens (IOL) implantation with conventional mode (Group A, n = 40) or torsional and burst mode (Group B, n = 40), using a computer-generated table.

### 2.2 Preoperative assessments

All the preoperative and postoperative assessments were performed by the same ophthalmologist, who was blinded to the procedure. The complete preoperative ophthalmic examination included uncorrected visual acuity, best-corrected visual acuity (BCVA), IOP, slit-lamp biomicroscopy, fundoscopic examination, ocular ultrasonography, central corneal thickness (CCT), corneal endothelial cell density (ECD), and macular optical coherence tomography. The corneal ECD was counted with a noncontact corneal endothelium microscope (NonconRobo Pachy SP 2000, Topcon, Tokyo, Japan). The CCT was recorded using the Visante optical coherence tomography system (Carl Zeiss Meditec, Oberkochen, Germany). Cataracts were evaluated with an IOL Master (Carl Zeiss Meditec, Oberkochen, Germany). For patients with severe media opacity, cataracts were evaluated using A-scan (Quantel Medical Cine Scan 150; Quantel Medical, Clermont-Ferrand, France).

### 2.3 Surgical technique

All the patients received levofloxacin eye drops 2 days before the surgery, and compound tropicamide eye drops 30 minutes before surgery to dilate the pupil. All the surgeries were performed by the same surgeon. The patient was placed under local anesthesia with topical oxybuprocaine eye drops, with the help of a ZEISS operating microscope (Carl Zeiss Meditec, Oberkochen, Germany) and Alcon Infiniti System (Alcon Laboratories, Fort Worth, Texas). A MicroTip 0.9-mm Aspiration Bypass System phacoemulsification tip (45°, flared Kelman) was used in the operation.

After a 3.2-mm self-sealing clear corneal incision was made, CCC was performed, with a capsulorhexis diameter of 5.0 to 6.0 mm. One drop of trypan blue (VisionBlue; Dutch Ophthalmic Research Center International, Zuidland, Netherlands) was injected to the anterior chamber through the side-port incision before CCC, if necessary. The quick chop technique was applied for nucleus fragmentation.

For patients treated with the conventional method (Group A), phacoemulsification with continuous mode was performed with ultrasound power 60%, vacuum limit 400 mm Hg, and aspiration flow rate 40 mL/min. Patients in the experimental group (Group B) were treated with combined torsional and burst mode, with torsional amplitude 60%, longitudinal ultrasound power 30% (burst; width 40 ms; off time 30 ms), vacuum limit 400 mm Hg, and aspiration flow rate 40 mL/min.

All eyes were implanted with an IOL (AR40e; Abbott Medical Optics Inc., Santa Ana, California) of 6-mm optical diameter, and then aspiration of Viscoat and hydration of the incision was performed. The patients were treated with subconjunctival injection of 0.5 mg dexamethasone and ofloxacin eye ointment on the conjunctival sac after the surgery. Postoperative Tobradex (0.3% tobramycin and 0.1% dexamethasone) eye drops and 0.1% pranoprofen eye drops were used for 2 to 4 weeks.

### 2.4 Measurements

The main phacoemulsification parameters were recorded, including the mean CDE and the mean UST. The BCVA, ECD, and CCT were measured 1 month postoperatively.

### 2.5 Statistical analysis

Statistical analyses were performed using the statistical package SPSS 10.0. The independent samples *t*-test was used to compare the differences in CDE and UST between the 2 groups. One-way analysis of variance was used to compare the postoperative changes in ECD and CCT between the 2 groups. The chi-squared test was used to compare the differences in postoperative BCVA. *P* values less than .05 were considered statistically significant.

## 3 Results

### 3.1 Clinical characteristics of patients

In Group A, 40 patients (40 eyes) were enrolled, including 23 men and 17 women (23 and 17 eyes, respectively; Table 1). The average age of these patients was 65.8 ± 4.7 years (range, 52 to 80 years). The preoperative BCVA fluctuated greatly among the population of Group A, including finger count, hand motion, BCVA 0.02 to 0.08, and light perception in 16, 11, 8, and 5 eyes, respectively. In addition, in Group A, the nuclear opalescence was Grade IV in 21 patients and Grade V in 19 patients.

**Table 1:**
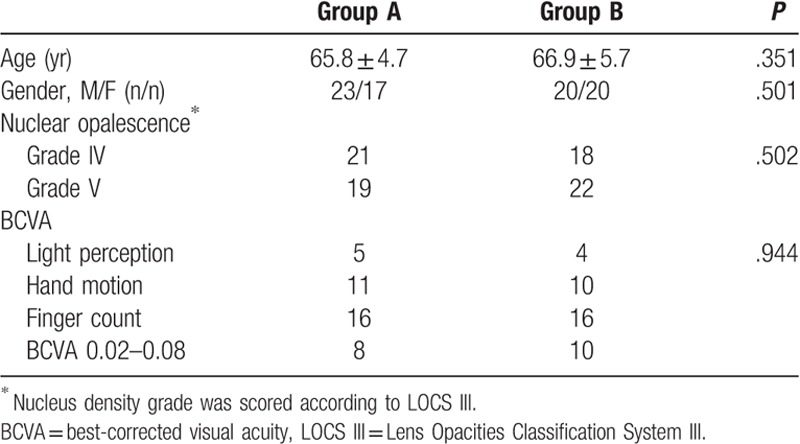
Demographics and clinical characteristics of the patients.

In Group B, 40 patients (40 eyes) were enrolled, including 20 men and 20 women (20 and 20 eyes, respectively; Table 1). The average age was 66.9 ± 5.7 years (range, 54 to 85 years). The preoperative BCVA included finger count, hand motion, BCVA 0.02 to 0.08, and light perception in 16, 10, 10, and 4 eyes, respectively. In Group B, the nuclear opalescence was Grade IV in 18 patients and Grade V in 22 patients.

The 2 groups were statistically comparable with regard to age, gender ratio, BCVA, and severity of nuclear density (Table 1).

### 3.2 UST and CDE

The UST of Group A (59.45 ± 14.36 seconds) was significantly greater than that of Group B (48.54 ± 17.82 seconds; *P* = .041; Table 2). The CDE of Group A (17.65 ± 6.24%) was significantly greater than that of Group B (14.64 ± 5.43%; *P* = .039).

**Table 2:**
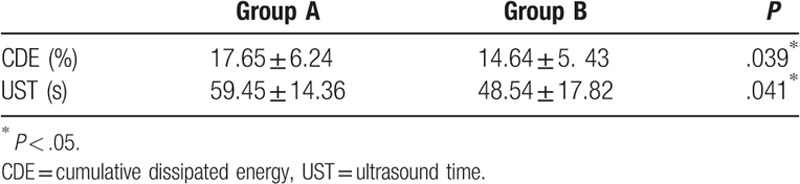
CDE and UST used during the operation.

### 3.3 Surgical complications

Posterior capsular rupture was the major intraoperative complication. Intraoperative posterior capsular rupture occurred in 1 (2.5%) patient in both Group A and Group B, which was not a significant difference (Table 3).

**Table 3:**
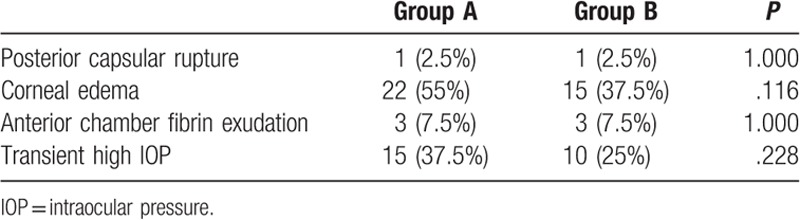
Surgical complications.

All postoperative complications were recorded within 4 days after surgery, including corneal edema, anterior chamber reaction and flare, high IOP, and pupil deformation. There was no occurrence of other complications, such as pupillary capture, IOL deviation, retinal detachment, or endophthalmitis. All the complications had resolved by 1 month after surgery. OCT examination at 1 month after surgery showed no cystoid macular edema in any patients.

Corneal edema and Descemet membrane folds occurred in 22 (55%) and 15 (37.5%) patients in Groups A and B, respectively. Both corneal edema and Descemet membrane folds disappeared within 2 to 4 days postoperatively after local application of tobramycin and dexamethasone eye drops and systemic corticosteroid treatment. Anterior chamber fibrin exudation was observed in 3 patients of each group and was absorbed within 1 week after topical application of tobramycin and dexamethasone eye drops and systemic corticosteroid treatment. Transient high IOP was found in 15 and 10 patients in Groups A and B, respectively (37.5% cf. 25%), and was relieved within 2 days after topical use of ocular hypotensive medications. At 1 month after surgery, all patients had transparent cornea, negative anterior chamber flare, correct IOL position, and normal IOP.

### 3.4 Postoperative BCVA

BCVA was improved after surgery in both the groups. Specifically, in Groups A and B, the postoperative BCVA was improved in 36 (90%) and 38 eyes (95%), respectively. Visual acuity was not improved in 4 eyes (10%) in Group A nor in 2 eyes (5%) in Group B. Postoperative fundus examination showed that in patients with the following diseases, there was no improvement in visual acuity: macular degeneration, diabetic retinopathy, high myopia retinopathy, or other retinal diseases. Postoperative BCVA ≥ 0.5 occurred in 16 and 20 patients in Group A and Group B, which was not a significant difference.

### 3.5 Postoperative ECD and CCT

The preoperative ECD was 2237 ± 262/mm^2^ in Group A, which was not significantly different from that of Group B (2272 ± 309/mm^2^; Table 4). Preoperatively, all the patients had a normal transparent cornea. The average preoperative CCT was 556 ± 27 μm in Group A, which was not significantly different from that of Group B (551 ± 29 μm).

**Table 4:**
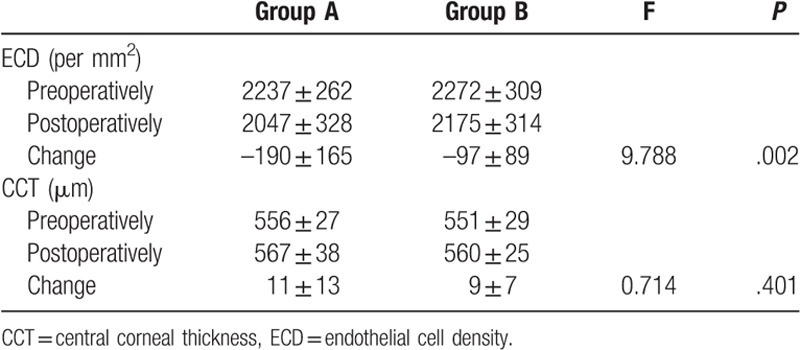
Preoperative and postoperative ECD and CCT preoperatively and postoperatively.

At 1 month after surgery, mild-to-moderate postoperative corneal endothelial cell loss and thickening of the CCT occurred in some patients (Table 4). The changes in corneal ECD at 1 month were significantly greater in Group A (−190 ± 165/mm^2^) than that in the Group B (−97 ± 89/mm^2^). The changes in the corneal CCT at 1 month were not significantly different between Group A (11 ± 13 μm) and Group B (9 ± 7 μm).

## 4 Discussion

Phacoemulsification and IOL implantation is the most popular cataract surgery.^[9]^ Phacoemulsification is used to remove the opaque lens with minimal ultrasonic power and surgical trauma. New surgical techniques have been developed to reduce the ultrasonic time and power during phacoemulsification, for better protection of the corneal endothelial cell. In the present study, we compared the torsional and burst mode with the conventional and determined that during the early postoperative period, the former was associated with less endothelial cell loss and better recovery.

The mature cataract with hard nucleus differs in ocular pathological and anatomical features from that of the soft nuclear cataract, with iris anterior synechiae and inelastic iris, and is associated with mydriasis difficulties. Many methods have been utilized to dilate the pupils, including viscoelastic substance sliding, equipment traction, pupil annular collar incision, iris longitudinal or full incision, and iris retractors.^[10,11]^ In the present study, in cases with a small pupil, we successfully performed these methods to dilate the pupil.

The anterior capsule of the mature or hypermature cataract is covered by a muddy or liquefied lens cortex, making it difficult to visualize and complicating the performance of CCC. In the present study, we injected trypan blue, which is the dye that is most used to stain the anterior capsule during capsulorhexis, with no red reflex into the anterior chamber. Although this procedure may increase the operative time, it can benefit capsulorhexis and subsequent manipulations greatly.^[12]^

The hard nuclear cataract often features the degeneration of zonules, lose of capsule, and cortical liquefaction, which have been associated with intraoperative complications such as posterior capsular rupture, vitreous prolapse, and even falling of the nucleus into the vitreous cavity.^[1,5]^ Postoperative inflammation in the hard nuclear cataract is usually more severe and of longer duration compared with the soft nucleus cataract. Therefore, it is recommended that topical mixed corticosteroid eye drops should be applied with higher frequency and longer duration in cases of hard nuclear cataract, and postoperative fundus examinations should be done regularly.

It has been reported that in phacoemulsification surgery, the torsional mode is more efficient than the conventional mode.^[13]^ The ultrasound power of the traditional phacoemulsification mode is produced from the longitudinal motion of the phacoemulsification needle, whereas the newer Ozil torsional mode has a different energy transfer. The ultrasonic frequency of the phacoemulsification tip in torsional mode decreases from 40 to 32 kHz, saving 20% of the ultrasound power and ensuring sufficient cutting efficiency.^[14]^ Unlike the longitudinal movement in the conventional mode, during removal of the lens nucleus in the torsional mode transverse vibrations of the probe are used. This results in a shearing motion and the phacoemulsification tip remains in constant contact with the nucleus^[15]^; the safety of heat transfer is improved greatly, lens nucleus rejection is eliminated, the emulsification area is large, and thiere is good nuclear follow-ability.^[13,16–18]^ The emulsification process also significantly reduces the traction force on the lens capsule and zonules, thus improving the safety and efficacy of the surgery.

Burst mode phacoemulsification is different from continuous mode and saves ultrasound power.^[19]^ The burst mode is characterized by instant high-energy releases. When a single nuclear blast occurs, the phacoemulsification needle is inserted into the core of the lens and causes complete obstruction. As the cavitation energy is relatively low, the vacuum rapidly rises to provide greater aspiration immediately, thus increasing the follow-ability and assisting the chopping process of the nucleus. The rebound strength of the burst mode phacoemulsification also is less than that of the nonburst mode, because of the high-speed vibration of the phacoemulsification needle. Thus, the total energy declines, making the effective phacoemulsification time much shorter.^[20]^ The lesser total ultrasound energy utilization during the operation is associated with more benefits including less injury to surrounding ocular tissue, less corneal edema, less endothelial cell loss, and rapid visual rehabilitation.

In the present study, we applied the torsional mode with burst to treat hard nuclear cataract. We evaluated the effect of cataract surgery on corneal endothelial cell injury using corneal ECD and CCT. We found that at 1 month after surgery, the torsional mode was associated with less change in the ECD compared with the conventional mode. Torsional phacoemulsification produces less heat, thus reducing heat-induced injury to the corneal endothelial cells.^[21]^ In addition, burst ultrasound mode can reduce the overall ultrasound energy, as evidenced by less mean CDE, and the effective UST in the torsional and burst mode compared with the conventional mode. As high ultrasound energy is associated with increased damage to corneal endothelial cells,^[4]^ the lower energy used in torsional and burst mode may contribute to less corneal endothelial cell injury. Our findings are consistent with the previous reports that the torsional burst phacoemulsification mode was associated with greater safety and less damage to the corneal endothelial cells compared with the conventional phacoemulsification mode.^[22,23]^

Damage of the corneal endothelial cells during cataract surgery positively correlates with the power and time of ultrasound.^[4]^ Burst ultrasound mode can reduce the overall ultrasound energy by reducing the mean CDE and the effective UST. Therefore, the combined torsional and burst mode has better efficiency than the conventional mode, and damage to the surrounding ocular tissue is less.^[5,24]^ In the present study, we found that the mean UST and CDE were significantly less than that of the conventional mode. However, at the 1-month postoperative timepoint, the conventional and experimental groups were statistically similar with regard to visual acuity and rate of capsular rupture and other intraoperative complications.

In conclusion, we found that the torsional burst mode combined with trypan blue staining was associated with less ultrasound energy utilization and less corneal endothelial cell injury, compared with the conventional. However, regardless of the surgical method used, phacoemulsification power should be kept at a minimum to induce minimal trauma and achieve early rehabilitation.

## Acknowledgments

This work was supported by the National Natural Science Foundation of China (No. 81300761) and Research Project of *Wuhan* Health and Family Planning Commission (WX18A08 and WX17Z02).

## Author contributions

**Conceptualization:** Wan-Ju Yang, Xing-Hua Wang, Fang Zhao, Zhong-Ming Mei, Shuang Li, Yi Xiang.

**Data curation:** Wan-Ju Yang, Xing-Hua Wang, Fang Zhao, Zhong-Ming Mei, Shuang Li.

**Formal analysis:** Wan-Ju Yang, Xing-Hua Wang, Zhong-Ming Mei, Yi Xiang.

**Funding acquisition:** Wan-Ju Yang, Xing-Hua Wang, Yi Xiang.

**Investigation:** Wan-Ju Yang, Xing-Hua Wang, Fang Zhao, Zhong-Ming Mei, Shuang Li, Yi Xiang.

**Methodology:** Wan-Ju Yang, Xing-Hua Wang, Fang Zhao, Zhong-Ming Mei, Shuang Li, Yi Xiang.

**Project administration:** Wan-Ju Yang, Xing-Hua Wang, Shuang Li, Yi Xiang.

**Resources:** Wan-Ju Yang, Xing-Hua Wang, Fang Zhao, Zhong-Ming Mei, Shuang Li, Yi Xiang.

**Software:** Wan-Ju Yang, Xing-Hua Wang, Fang Zhao, Zhong-Ming Mei, Yi Xiang.

**Supervision:** Wan-Ju Yang, Xing-Hua Wang, Shuang Li, Yi Xiang.

**Validation:** Wan-Ju Yang, Xing-Hua Wang, Fang Zhao, Zhong-Ming Mei, Shuang Li, Yi Xiang.

**Visualization:** Wan-Ju Yang, Xing-Hua Wang, Fang Zhao, Zhong-Ming Mei, Shuang Li, Yi Xiang.

**Writing – original draft:** Wan-Ju Yang, Xing-Hua Wang, Fang Zhao, Zhong-Ming Mei.

**Writing – review & editing:** Wan-Ju Yang, Xing-Hua Wang, Fang Zhao, Zhong-Ming Mei, Shuang Li, Yi Xiang.
